# Suitability and Sensitivity of Golden Grey Mullet *Chelon auratus* (Risso, 1810) as a Reference Fish Species for Ecotoxicity Tests in the Black Sea

**DOI:** 10.3390/toxics10050222

**Published:** 2022-04-28

**Authors:** Victor Niță, Magda Nenciu, Valentina Coatu

**Affiliations:** 1 Marine Living Resources Department, National Institute for Marine Research and Development “Grigore Antipa”, 300 Mamaia Blvd., 900581 Constanta, Romania; vnita@alpha.rmri.ro; 2 Chemical Oceanography and Marine Pollution Department, National Institute for Marine Research and Development “Grigore Antipa”, 300 Mamaia Blvd., 900581 Constanta, Romania; vcoatu@alpha.rmri.ro

**Keywords:** protocol, ecotoxicity, acute toxicity, golden grey mullet, sensitivity, reference species

## Abstract

In recent years, hydrocarbon exploration and production operations have intensified in the Black Sea. Alongside growth in exploration and production activities, the influence of chemical usage across multiple industrial sectors within the Black Sea environment has become increasingly interesting. The aim of this research was to define a protocol for determining the acute toxicity of chemicals using the golden grey mullet, *Chelon auratus* (Risso, 1810), a native pelagic fish species of the Black Sea. Juvenile golden grey mullets were exposed for 96 h, under semi-static conditions, to dilutions of the reference toxicant 3,5-Dichlorophenol. Results from three reference toxicity tests (LC_50_ = 1.25 mg/L, 1.739 mg/L, and 1.409 mg/L) indicated that *C. auratus* is of moderate sensitivity when compared to literature values from EPAs Ecotox database. The protocol described within is intended to ensure Black Sea native organisms are represented by standard hazard assessment practices.

## 1. Introduction

Aquatic toxicology, broadly defined as the study of the effects chemicals or materials from natural or anthropogenic sources have on aquatic organisms [1], has played an important role in describing the influence of xenobiotic releases into the environment. As such, aquatic organisms have been used as early warning and monitoring systems for pollutant loads [2]. Toxicity testing is performed to identify the degree to which chemicals can damage living organisms in a controlled environment and has several major objectives: to obtain toxicity data for various chemicals, to aid in estimating and managing risks posed by various toxicants, to aid in setting chemical regulations and environmental standards, and ultimately, to classify chemicals based on how toxic they are to various species [3].

Toxicity studies in aquatic species have not only documented the susceptibility of individual species to a wide range of toxicants, but also served to highlight several fundamental principles, such as bioaccumulation within the individual organism and biomagnification along the food chain [2].

The principle upon which toxicity tests are based is the recognition that the response of living organisms to the exposure of external stressors (chemical or biological) is dependent upon the dose of the stressor [4]. Using this principle, aquatic toxicity tests are designed to describe a concentration–response relationship, where the measured effect is plotted graphically with the concentration.

Early toxicity tests lacked standardization and varied widely in environmental and chemical exposure conditions. This variation in test conditions led to ineffective interpretation of chemical hazard [1]. Efforts to standardize aquatic tests were performed in the 1970s and the United States Environmental Protection Agency (US EPA) sponsored a dedicated workshop that resulted in a document describing the standard methods for acute toxicity testing for fish and invertebrates [5]. This important publication has been the primer for subsequent aquatic standards development and has been used worldwide.

Acute toxicity tests are usually designed to evaluate the concentration–response relationship for survival, whereas chronic studies evaluate sublethal effects such as growth, reproduction, behavior, tissue residues, or biochemical effects and are usually designed to provide an estimate of the concentration that produces no adverse effects [1]. Acute toxicity tests are short-term tests designed to measure the effects of toxic agents on aquatic species during a short period of their life span. Acute toxicity tests evaluate effects on survival over a 24 to 96-h period. The American Society for Testing and Materials (ASTM) [6], Environment Canada (EC) [7], and the US EPA [5,8,9] have elaborated and issued standard guides on how to perform acute toxicity tests for pelagic and benthic species for both freshwater and marine invertebrates and fishes. 

Generally speaking, species selection for toxicity testing must consider several different factors, including size, ease of maintenance in the laboratory, convenience for testing, relevant economic, biological or ecological factors, known sensitivity, pre-existing data, animal welfare, availability of test methods for subsequent tests that may be triggered, as well as national or regional preferences. There are also practical considerations, such as the availability of cultured, as opposed to field-collected organisms [10]. Species selection depends on regulatory requirements and on environmental exposure scenarios (cold, temperate or warm water species, freshwater or estuarine/marine fish) [11]. Species which have been described and standardized for environmental hazard assessment are preferred (e.g., *Danio rerio, Pimephales promelas, Menidia beryllina, Cyprinodon variegatus*) [12,13]; however, hazard assessment in new regions may require using novel organisms to describe region specific organism sensitivity interests.

Novel species selection must follow standard criteria to be considered amenable for laboratory conditions. In these cases, a characterization of the organism and the site from which they are collected should be undertaken. The characterization should include an assessment of the contamination history of the collection site, evidence that the animals are derived from a viable population (i.e., reproducing), and their parasite load. Once in the laboratory, acclimation of the population to laboratory conditions should include mortality, disease, and stress assessment [10]. When new species are identified as candidates to be used in hazard assessment, the relative sensitivity against standard laboratory test organisms should be assessed to facilitate a stronger understanding of how the new species will be used to inform hazard and risk assessment efforts.

In recent years, interests and development of oil and gas exploration and production have intensified in the Romanian Black Sea [14]. The Romanian offshore area covers 22,000 square km and reaches depths beyond 1000 m [15]. The whole area is divided in blocks of different sizes, with some being awarded to operators for exploration, development, and production activities. Current exploration continues in shallower water (depth up to 50 m), but there are interests to develop new plays represented by slope and basin-floor fans located farther offshore [16,17]. In this context, there is a growing interest to investigate whether chemicals used during oil and gas production have any impact on the Black Sea environment. To manage environmental risks associated with oil and gas activities, globally accepted environmental hazard and risk assessment practices (Offshore Chemical Notification Scheme-OCNS [18], National Pollution Discharge Elimination System-NPDES [19], Directive 2008/105/EC) [20] are implemented to regulate standard operational discharges through chemical selection, waste management, and environmental monitoring processes. Under such circumstances, establishing allowable limits for effluent discharge in the oil and gas industry is essential. For this, relevant and reliable reference species are required for ecotoxicity testing. Whereas none of the standardized fish species are found in the Black Sea, we selected the golden grey mullet *C. auratus* as the most suitable. Despite the fact that the test species is not in the list included in OCSPP 850.1075: *Freshwater and Saltwater Fish Acute Toxicity Test* [9]; it is widely spread in the Black Sea basin [21], easily adaptable to laboratory conditions and, if necessary, it can be bred in captivity, which makes it a suitable reference species to be used with this ecotoxicity testing protocol. None of these characteristics apply to other small pelagic species in the area (such as *Sprattus sprattus* (Linnaeus, 1758)-sprat-and *Engraulis encrasicolus* (Linnaeus, 1758)-anchovy). Moreover, mullets are documented for being used in chemical and toxicity testing worldwide starting from the 1980s to date [22,23,24,25,26,27].

The primary objective of our research was to establish a protocol for determining the sensitivity of native Black Sea organisms using acute toxicity testing of a reference toxicant for a native pelagic fish species (namely the golden grey mullet *C. auratus*) by calculating the lethal concentration resulting in 50% mortality of the exposed fish (LC_50_). The secondary objective of this research was to assess the sensitivity of the species by comparing LC_50_ results for the golden grey mullet to reported literature and guideline values (resulting in a species sensitivity distribution (SSD) curve), in an effort to reduce reliance on novel species and facilitate transition to standard laboratory test species for ease of future hazard assessment.

## 2. Materials and Methods

The current protocol was developed through review of existing fish toxicity test guidelines, with focus given to the OCSPP Guidelines 850.1075: Freshwater and Saltwater Fish Acute Toxicity Test [9], OECD Fish Toxicity Testing Framework [10], and OECD Test Guideline No. 203 Fish, Acute Toxicity Testing [11]. The provisions of these guidelines were adapted to the unique requirements of *C. auratus* and to local conditions in the Black Sea. The study was conducted according to the guidelines of the Romanian legislation on the protection of animals used for scientific research (Law no. 43/2014) and approved by the Ethics Committee of the National Institute for Marine Research and Development “Grigore Antipa” Constanta (protocol code no. 1 of 3 February 2022).

### 2.1. Laboratory Equipment

Normal laboratory equipment is necessary for the conduct of this assay [11]. Glass or other chemically inert containers should be used as test vessels. The dimensions of the vessels should be large enough to keep fish free of stress and the number of fish placed in each test vessel should not be so large as to cause the dissolved oxygen concentration to fall below the recommended levels or affect the results for the test. Fish loading should not exceed 0.8 g wet weight of organism per liter (g/L) [9]. Test vessels should be randomly positioned in the test area and shielded from unwanted disturbance (excessive noise, vibration, light variation).

### 2.2. Organism Selection and Acquisition 

Golden grey mullet *Chelon auratus* (Risso, 1810) was considered the primary test species, with big-scale sand smelt (*Atherina boyeri* Risso, 1810) as a secondary test option (subject to availability at the time of testing). Golden grey mullet juveniles were collected from the wild and acclimated to laboratory conditions following the recommendations below. Under current timeline restrictions, it was not possible to obtain these fish by controlled breeding in the laboratory. However, golden grey mullets can be bred in captivity [28,29], in order to provide a reliable batch of fish for experimental use [30].

Fish should be juveniles and originate from the same source and population to ensure uniformity. The fish should be of the same age (if unknown, it can be estimated via the size) and have normal appearance [11]. Based on the natural reproduction period documented for the sampling location and on expert judgement, the fingerlings shall be between three and nine months of age at initiation of exposure.

Juvenile fish weighing <3.0 g and being old enough to be actively feeding should be tested. The wet weight and length of at least 1% of the individuals from the batch of fish used in a particular test were measured and the mean values and ranges subsequently reported. The sizes within the batch will be such that longest fish should not be more than twice the length of the shortest fish [9].

Collected juvenile organisms should be immediately transported to the laboratory in aeration containers and acclimated in holding tanks made of glass, fiberglass, or other relevant aquaria material with natural seawater. Captured wild organisms must be quarantined immediately and monitored for a certain period to ensure organism health. The recommended holding period is 14 days. A minimum of 7 days of this period are used for acclimation to environmental conditions (e.g., temperature, light intensity, temperature, dilution water) similar to those used in the test. To maintain organisms in good condition and avoid unnecessary stress, they should not be crowded or subjected to rapid changes in temperature or water quality [9]. Following a 48-h settling-in period, mortalities should be recorded, and the following guidelines should be applied: mortalities of greater than 10% of the population in the 7 days of acclimation: rejection of the entire batch; mortalities of between 5 and 10% of the population during the 7 days of acclimation: acclimation continued for an additional 7 days; mortalities of less than 5% of the population during the 7 days of acclimation: acceptance of batch. Dead individuals must be immediately removed from the tank and remaining detritus extracted by siphoning [9].

Regarding health status monitoring, fish should not be used for a test if more than 5% of the culture or acclimating group dies or shows signs of stress (e.g., disease, physical damage, or abnormalities) during the 48 h preceding the test; if they have been used in a previous test, either in a treatment or in a control group; or if disease treatments were administered within 48 h of test initiation. Fish should not receive treatment for a disease during a test [9]. Fish should not be displaying visible signs of disease and stress and should be free of any apparent malformations and not have been previously treated against disease or parasites within the last 14 days prior to testing [10]. After 1 day of acclimation, the fingerlings are to be fed with JBL NovoGranoColor mini pellets (2% of fish biomass) [9]. No feeding shall be undertaken 48 h prior to and during the experiments.

### 2.3. Test Check-List

Test conditions must be appropriately documented, at test initiation and termination of the study along with the test procedure used (flow-through, static, or semi-static) [9]. Dilution water source and chemical characteristics (pH, salinity, temperature, and dissolved oxygen), as well as the preparation method of test solutions, frequency of renewals, and concentrations used shall be detailed. The test design (e.g., number of replicates, number of adults per replicate), the description of the test chambers, volume of solution, and information on feeding and handling techniques are compulsory [9].

### 2.4. Parameter Monitoring 

Temperature and light (intensity and photoperiod) should be actively monitored throughout testing. Prior to test initiation and every 24 ± 2 h thereafter, pH, salinity, dissolved oxygen, and temperature will be monitored per concentration. It is also recommended to measure inorganic nitrogen and total organic carbon (TOC). The photoperiod shall be 16:8 or 12:12 h light:dark. Light intensity will range from 540 to 1080 lx. The dissolved oxygen concentration should be between 60 and 100% saturation during the test and, for the 5 L test jars used, we achieved this by providing gentle aeration (1–2 bubble per second from a 10 mL glass pipette tip submerged to the bottom of the test chamber) [9]. Regarding water pH and salinity, they should be within the normal range of the test species habitat; in the case of the western Black Sea shelf, this should be 8.1 ± 1 pH and 11-18 PSU salinity, respectively [31].

### 2.5. Data Processing and Calculation of LC_50_

LC_50_ was the statistical estimate for the concentration in the medium necessary to result in 50% mortality from the test population and was calculated using the AAT Bioquest. Quest Graph™ LC_50_ Calculator [32] (no confidence interval provided by the software). For each concentration, the data from the replicates should be pooled to calculate the mortality percentage after 24 h, 48 h, 72 h, and 96 h in relation to the total number of fish tested. The 96-h LC_50_ should be determined by an appropriate statistical method (sigmoid function) [32]. In cases where the data are insufficient, the minimum concentration corresponding to 100% mortality and the maximum concentration corresponding to 0% mortality shall be reported [9]. If mortalities occur in the control, the Abbot formula for the correction of natural mortality should be applied [33].

### 2.6. Development of the Species Sensitivity Distribution (SSD) Curve

In an effort to relate the results from the reference toxicity assays, the EPAs ECOTOX database was queried for acute aquatic toxicity data (LC_50_, EC_50_) associated with 3,5-Dichlorophenol. The resulting dataset (taxa and stressor intensity), along with the LC_50_ data from the current study, were used to generate a species sensitivity distribution (SSD) using the EPAs Species Sensitivity Distribution Generator [34,35,36,37]. Overall, the ECOTOX dataset used to describe 3,5-Dichlorophenol toxicity is limited; however, it does provide insight into the potential sensitivity of *C. auratus* in relation to other test organisms. HC5 (hazardous concentration for 5% of the species) and the stressor intensity for *C. auratus* were also calculated.

## 3. Results and Discussion-Protocol Design 

This protocol was optimized after performing three reference toxicant tests to benchmark toxicity on Black Sea native species (golden grey mullet), at an exposure temperature of 20 °C. All the steps necessary in order to apply the protocol are detailed below.

### 3.1. Species Selection

As mentioned above, the selected test species was the golden grey mullet [*C. auratus* (Risso, 1810)], as representative native pelagic fish of the Black Sea, with a size and handling tolerance appropriate for laboratory study.

### 3.2. Fish Collection and Acclimation

Acclimation procedures are separated into two distinct stages. The first stage is fish acclimation to standard laboratory conditions (this can be ignored if not using wild-caught organisms), while the second stage is conditioning to experimental conditions.

**Figure 1:**
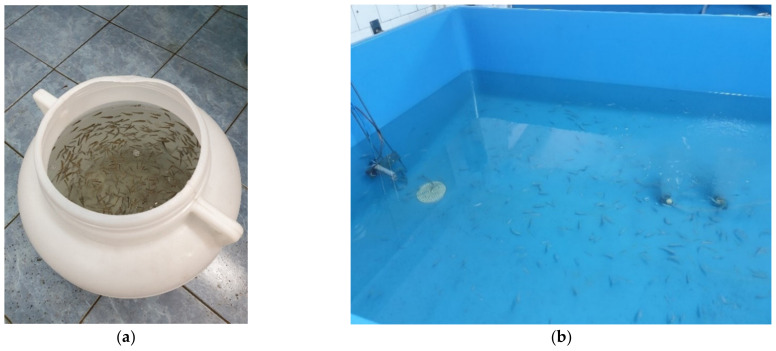
(**a**) Juvenile golden grey mullets transported to the laboratory in 40-L barrels; (**b**) Fish batch acclimation in 900-L FRP tanks in NIMRD’s flow-through system (original photos).

Juvenile golden grey mullets (mean length 1.8 ± 0.5 cm, mean biomass 0.5 ± 0.1 g, aged 5–7 months) were captured from the wild, near Constanta, Romania (GPS 44°13′9.74″ N, 28°38′58.31″ E), using mesh nets (Mivardi Rubber, 3 mm mesh size), subsequently placed in 40-L polypropylene barrels (Sterk Plastic), and transported immediately to the laboratory (Figure 1a). During the transfer, aeration was provided using Hailea or Hydor air pumps. The fish were gently transferred from the barrels to the tanks, making sure that the temperature difference between barrels and tanks was ≤3 °C. In such a case, where temperature is >3 °C, temperature should be equalized by mixing small amounts of water into the transportation containers until the temperature between the two containers falls within the 3 °C limitation. The experimental batch was kept in the 900-L fiberglass-reinforced plastic (FRP) tanks using a flow-through system (having a flow rate of 5 L/min) with a water turnover rate of 3 h for a minimum of 14 days, to ensure proper acclimation to laboratory conditions. Wild-caught fish should be given considerable time to recover from disturbances (Figure 1b).

Throughout the acclimation period, the water was UV sterilized at a flow of approximately 100 L/h and proper aeration was provided, consisting of an air flow of approximately 300 L/h, in order to obtain around 80–90% dissolved oxygen (DO) saturation. 

The fish were fed daily with JBL NovoGranoColor mini pellets, at an approximate ration of 2% of total fish biomass in the tank [38] (average measurements were performed based on the technique described in Section 2.2 and extrapolated).

### 3.3. Fish Conditioning

Conditioning, the second stage of the acclimation procedure, is the process in which test fish are acclimated from standard laboratory conditions to desired experimental conditions [9]. The equipment and materials used included 50-L glass aquaria, laboratory fish nets (mesh size 1 mm), suitable-size fish pellets (JBL NovoGranoColor mini), an aeration pump (Hailea 200 W), and a JBL CristalProfi external filter.

**Figure 2:**
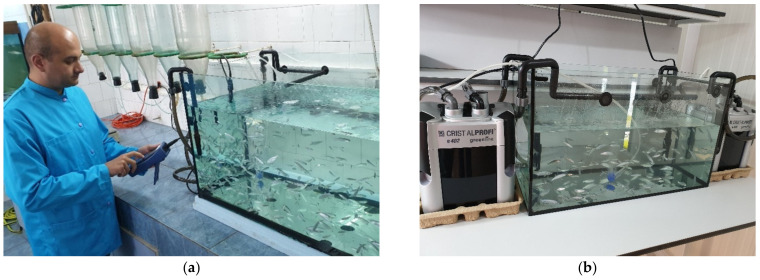
(**a**) Fish batch extracted from the FRP tank and transferred to the 50-L aquarium; (**b**) Experimental lot in the temperature-controlled room prior to test initiation (original photos).

Prior to the initiation of each reference toxicity test, the required number of fish needed for the experiment +10% maximum allowed mortality rate were extracted from the fish pool and placed in the 50-L aquarium (Figure 2a). Fish health was continuously monitored and any fish showing signs of stress or disease [39] were removed from the tank and euthanized according to the procedure explained below [40].

The experimental batch of test organisms was progressively adapted to test conditions by modifying the controlled room temperature where the experimental batch was placed, to reach the testing temperature (20 ± 1 °C) (Figure 2b), but not by more than 1.5 °C each day [9]. The number of days required for temperature acclimation was calculated before scheduling each experiment. Aeration was provided, an air flow of about 50 L/hour, in order to obtain around 80–90% DO saturation, as well as water recirculation and filtration. The daily amount of food was progressively reduced, from a ratio of 2% of total fish biomass in the tank to 1%, in the first few days [41]; 48 h before testing, no more food was provided, so that fish excretion would not influence water parameters in the test jars.

### 3.4. Dilution Water Preparation and Oxygenation 

The dilution water used was natural Black Sea water pumped from a pollution-free location off NIMRD’s premises and stored in an underground 130 m^3^ decanter, which was analyzed for the presence of contaminants for experiments/testing use. The seawater reaches the aquaculture laboratory gravitationally, through a pipeline system. Once in the laboratory, seawater was analyzed before each running test to confirm the ambient conditions of the receiving waters and no contamination. The following parameters were measured: temperature, pH, salinity, dissolved oxygen, nutrients, pollutants (hydrocarbons, persistent organic pollutants, and heavy metals), using a Mettler Toledo Seven Excellence multiparameter probe and internationally agreed seawater analysis methods [42,43,44,45].

Subsequently, to ensure that the dissolved oxygen saturation content of the dilution water was between 90% and 100% prior to use for fish tests, DO was measured before initiating the testing procedure. Whenever DO saturation was less than 95%, additional aeration for 10–15 min was provided using the air pumps. When reaching levels between 95% to maximum 100% DO saturation, the aeration process was stopped, and the storage tanks were sealed and stored in the temperature-controlled room at test temperature. The water in the tanks was used the following day for test substance dilution preparation.

### 3.5. Fish Randomization

**Figure 3:**
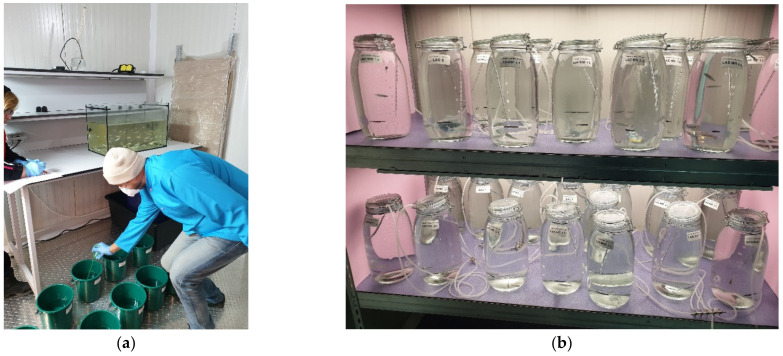
(**a**) Fish batch randomization using plastic buckets; after reaching the desired number [4] of individuals per bucket, the contents of each bucket were strained through a net and all fish were transferred to the matching labeled experimental jar; (**b**) Experimental jars randomized on the rack in the temperature-controlled room (original photos).

Fish were randomly distributed in the experimental jars, as it is the most reliable method for creating homogeneous treatment groups, reducing potential biases or judgments [46]. The equipment and materials used during the transfer of test organisms from holding containers to test aquaria included labeled 5-L plastic buckets containing 2 L of seawater (Figure 3a), a small net for extracting the fish, labeled experimental 4-L glass jars filled with the exposure solution, and a randomization block scheme obtained using Random Lists software [47], both for distributing the fish in the buckets and arranging the position of test jars on the rack (Figure 3b).

For the randomization procedure, the fish were individually extracted from the aquarium in the temperature-controlled room using the fish net, and, following the design of the randomization scheme, placed one by one in the plastic buckets. When reaching the desired number [4] of individuals per bucket, the contents of each bucket were strained through a net and all fish were transferred to the matching labeled experimental jar, in order to avoid uneven exposure times. After a thorough visual inspection of the jars and confirmation of conformity (number of fish/jar), the jars were placed on the experimental rack according to a randomized set-up. Copies of the randomization schemes were kept in the raw data files for all experiments.

### 3.6. Acute Toxicity Testing

#### 3.6.1. Reference Substance

The reference toxicant used for tailoring the protocol was 3,5-Dichlorophenol (CAS# 591-35-5), a substance commonly used as a reference substance to act as a positive control [48]. The concentrations applied to acute toxicity testing on fish were determined according to reference values defined by ISO standards 10253 [49] and 14699 [50], though no standard directly references 3,5-Dichlorophenol as a positive control substance for acute fish testing. The laboratory was simultaneous developing methods for *Acartia tonsa* and *Skeletonema costatum*. In an effort to standardize reference toxicity testing for the laboratory, one test substance (3,5-Dichlorophenol) was applied across all testing platforms. Concentrations identified for *C. auratus* reference testing were defined based on ISO standards described above and reported LC_50_ literature values ranging from ~1–3.5 mg/L [51,52,53,54,55]. An initial range-finding test was conducted at a broader concentration range, as not all literature values were in agreement [51].

#### 3.6.2. Experimental Design

The golden grey mullet 96-h acute toxicity testing was performed in 5000 mL glass jars containing a total volume of 5000 mL of solution. Each jar was covered by a glass lid, in order to avoid contamination, evaporation and escape of the test organisms. Each test jar was labeled with the species initials, treatment group and replicate. Aeration was provided in the jars in order to maintain a DO level higher than 60% [9]. After setting-up the jars according to the randomized design, light intensity was measured using a Delta OHM HD 2302.0 light meter and adjusted between 540 to 1080 lx, as indicated by the US EPA guidelines [9].

#### 3.6.3. Test Substance Preparation and Administration

The stock solution for 3,5-Dichlorophenol was prepared on the day of testing by adding 1 g of 3,5-Dichlorophenol to 1 L of natural seawater in a 1 L glass volumetric flask and stirred on a magnetic stirrer for 15 min at 700 rpm. Natural seawater was used as a control medium and as dilution water.

**Figure 4:**
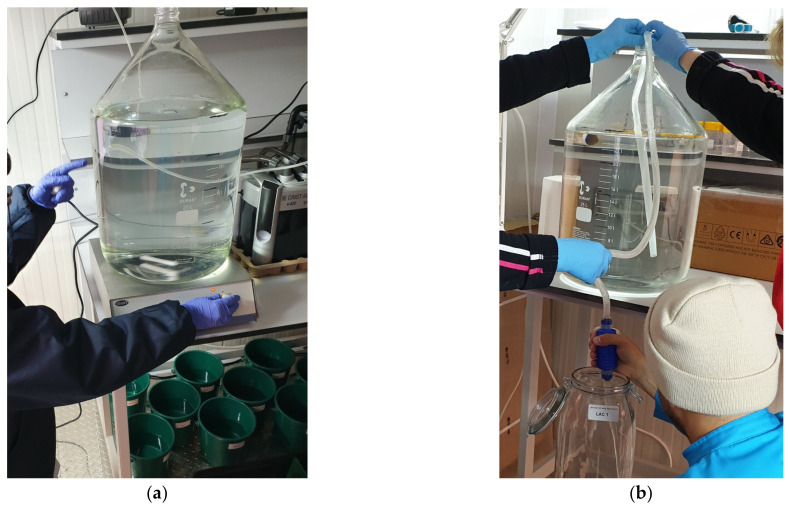
(**a**) Homogenization of the toxicant solution on a magnetic stirrer; (**b**) Distribution of the solution in the experimental jars (original photos).

A series of 5 dilutions (0.2, 0.4, 0.8, 1.6, and 3.2 mg/L) were prepared at using the 1 g/L stock solution by diluting the stock solution with natural seawater, in 25,000 mL glass carboys labeled accordingly, from 1 to 5 (Table S1). Each solution was homogenized by a magnetic stirrer for 15 min at 700 rpm (Figure 4a), after which it was distributed into 5000 mL volumes in the experimental jars (Figure 4b). 

Every 24 h, 80% of the tested solution was renewed using freshly prepared stock and dilution solutions. Renewal occurred by siphoning and replacing 80% exposure solutions from each replicate vessel (Table S1).

All resulting wastewater was stored in closed barrels labeled accordingly (“hazardous waste”) and disposed of by a specialized company.

#### 3.6.4. Parameter Monitoring

Temperature, pH, salinity, dissolved oxygen, and inorganic nitrogen were measured at the beginning of the tests, before the renewal (new medium), and at the end of each 24-h exposure period (old medium) for each replicate and concentration (Table S2.1–S2.3), using a Mettler Toledo Seven Excellence multiparameter probe and internationally agreed seawater analysis methods [42,43,44,45].

### 3.7. Mortality Observation and Euthanasia

Observations on fish mortality and immobilization were performed and recorded at 24 ± 1 h intervals after the start of the test. Immobilization was considered as the lack of swimming ability [9]. Dead fish were removed at each observation interval and stored in labeled zip-lock bags in a deep freezer.

**Figure 5:**
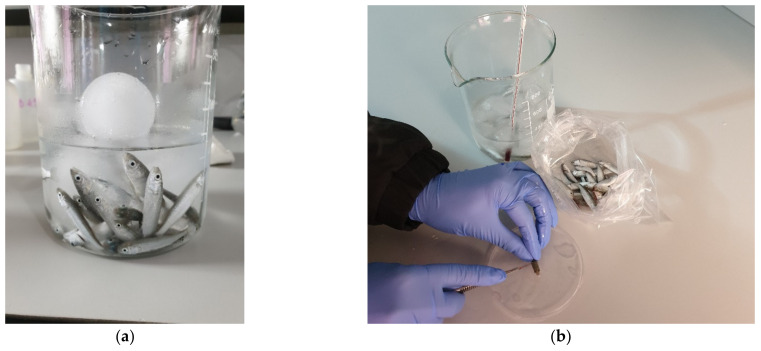
(**a**) Fish placed in ice slurry for euthanasia; (**b**) Sectioning the spinal cord (original photos).

After the 96-h test period, the surviving fish were euthanized humanely, in compliance with the American Veterinary Medical Association Guidelines for the Euthanasia of Animals [40]. The fish from each jar were strained through a net and placed in a 1 L glass jar filled with ice slurry (Figure 5a). No additional anesthetic was used. When observing complete immobility, the fish were extracted individually, and the spinal cord was gently sectioned using a sharp scalpel (Figure 5b). Upon completion, the euthanized fish were put in labeled zip-lock bags and stored in a deep freezer until they were collected for disposal by a specialized company.

### 3.8. Data Processing and Interpretation

For each concentration, data from each replicate were collected and the mortality rate was calculated after 24 h, 48 h, 72 h, and 96 h relative to the total number of fish used. The LC_50_ at 96 h was calculated using a sigmoid function with the LC_50_ calculation application from AAT Bioquest [32] (Table S3).

### 3.9. Significant Results: LC_50_ Values

The first test was treated as a range-finding study, using a relatively broad dose range (control and 1.25, 2.5, 5, 10, and 20 mg/L) to improve confidence in final dose selection for subsequent tests. The range finding test did not meet minimum LC_50_ calculation requirements (at least two assay concentrations whose predicted response is less than 50% and two whose predicted response is greater than 50%) [56]. Additionally, this test experienced excursions from dissolved oxygen and light intensity requirements. Despite the test not meeting performance criteria, it did provide sufficient evidence to define a narrower dose range for subsequent tests (control, 0.2, 0.4, 0.6, 1.6, and 3.2 mg/L). For the subsequent tests, critical issues were corrected, so as to comply with the guidelines [9]. No mortalities were recorded in the control replicates (thus, no Abbot formula application was required) [33]; consequently, the tests were considered valid.

**Figure 6:**
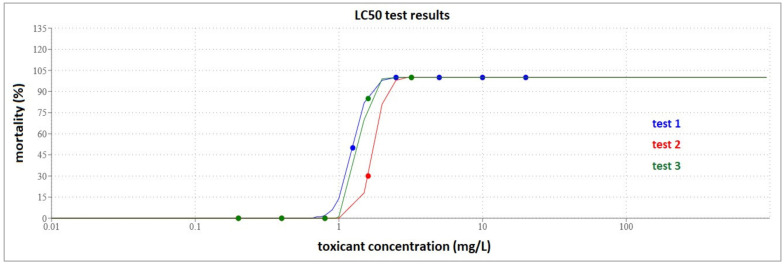
LC_50_ calculation for the reference toxicant tests 1, 2, and 3 (LC_50_ = 1.250 mg/L, 1.739 mg/L, 1.409 mg/L).

The LC_50_ results obtained from all test runs (1.25, 1.739, and 1.409 mg/L) were considered relevant, being in the expected range of 1–4 mg/L (Figure 6) [56]. However, the test results from the first test (LC_50_ 1.25 mg/L) were excluded from species sensitivity distribution (SSD) calculations due to potential confounding stress from inadequate dissolved oxygen during testing. During all tests performed, temperature, salinity, and pH in the dilution water varied within the range of the Black Sea (Table S2.1–S2.3) [31]. 

At test initiation and before renewal (new), NO_2_, NO_3_, and NH_4_ were considered to be represented by the dilution water. All inorganic nitrogen values were below the maximum allowable concentration in Romania [57]. At the end of each 24-h exposure period, inorganic nitrogen was measured, and, during each test, ammonium increased after 24 h, 48 h, 72 h, and 96 h, within the same variability range (Table S2.1–S2.3). The high levels of NH_4_ in old dilutions are probably caused by fish metabolism, which determines excretion products to accumulate, as the system was semi-static. Taking into consideration the fact that similar values were also found in control jars, where no effect was observed; it was considered that NH_4_ levels did not cause any mortality during testing, but the high levels of reference toxicant did.

### 3.10. Sensitivity of C. auratus in Relation to Literature Data

**Figure 7:**
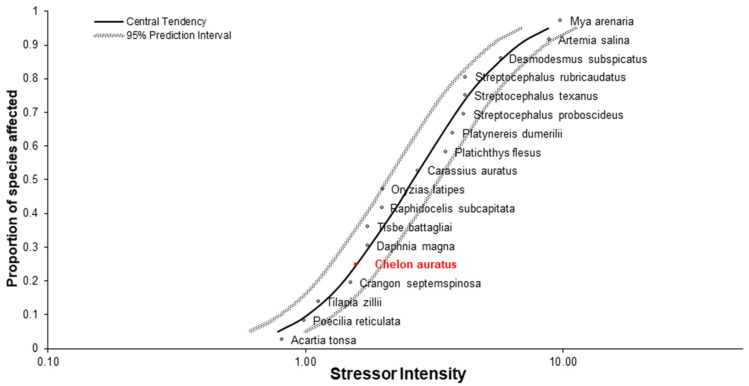
Species sensitivity distribution calculation: golden grey mullet included amongst a range of aquatic test organisms to demonstrate general sensitivity as related to a broad range of aquatic organisms.

In an effort to relate the results from the reference toxicity assays, data from EPA’s ECOTOX database [34,35,36,37] along with the calculated LC_50_ values were used to generate a species sensitivity distribution (SSD) (Figure 7). Overall, the ECOTOX dataset used to describe 3,5-Dichlorophenol toxicity is limited; however, it does provide insight into the potential sensitivity of *C. auratus* in relation to other test organisms. The SSD indicates *C. auratus* is likely of moderate sensitivity, HC5 = 0.78 (0.61–1.0) and the stressor intensity for *C. auratus* = 1.6 (1.53–1.7), if not slightly more sensitive than standard laboratory test species to the test substance, 3,5-Dichlorophenol. This indication of sensitivity requires further testing to confirm, as there is a paucity of toxicity data associated with 3,5-Dichlorophenol. Additional assays directly comparing the sensitivity of *C. auratus* to *Danio rerio* or *Cyprinodon variegatus* are recommended be conducted prior to any novel fish species being adopted as standard laboratory test organisms for toxicity assessment.

## 4. Conclusions

The protocol for using the golden grey mullet *C. auratus* as a suitable reference fish species for ecotoxicity tests in the Black Sea proved to be applicable and provides significant support for testing the environmental impact of various xenobiotics (related, but not limited to the oil and gas industry) introduced in the environment.

The primary aim of our research was to set up a protocol for assessing the acute toxicity on pelagic fish species, applicable at regional level, using the golden grey mullet as a native species representative of the Black Sea ecosystem. The selected species showed good adaptability to laboratory conditions, as well as an appropriate size for this type of testing. Juvenile golden grey mullets were exposed for 96 h, under semi-static conditions, with a daily renewal of 80% of the exposure medium, to a series of dilutions of the tested toxicant, prepared by adding the appropriate volume of stock solution in 1000 mL calibrated flasks labeled C0 to C5, filled with natural seawater and well mixed. Five replicates were prepared for each concentration and four organisms were distributed per replicate following a random design.

The final results (LC_50_ of 1.25 mg/L, 1.739 mg/L, and 1.409 mg/L) were calculated using a sigmoid function on mortality data collected throughout the study, and were in line with values reported in other fish species, ranging from 1 to 3.5 mg/L. The tests performed were considered acceptable from the fish survivability perspective, as control mortality did not exceed 20%. The only minor issues that appeared, related to dissolved oxygen concentrations outside the range indicated by the guidelines, were settled by providing proper aeration to the dilution water, as well as in the test jars. 

Moreover, a species sensitivity distribution (SSD) analysis was performed, which indicates that *C. auratus* is likely of moderate sensitivity (HC5 = 0.78 (0.61–1.0) and the stressor intensity for *C. auratus* = 1.6 (1.53–1.7)), if not slightly more sensitive than standard laboratory test species to the test substance, 3,5-Dichlorophenol. This indication of sensitivity requires further testing to confirm, as there is a scarcity of toxicity data associated with 3,5-Dichlorophenol. We recommend additional assays directly comparing the sensitivity of *C. auratus* to *D. rerio* or *C. variegatus* be conducted prior to any novel fish species being adopted as standard laboratory test organisms for toxicity assessment. Future work needs to be conducted to continue to benchmark the golden grey mullet against standard laboratory test species. Though novel organisms are of high value to specific regions, having the ability to relate novel organism to standard species facilitates extrapolation to other regions as well as standardizes testing across platforms. 

## Data Availability

Not applicable.

## Supplementary Materials

The following supporting information can be downloaded at: https://www.mdpi.com/article/10.3390/toxics10050222/s1, Table S1. Dilution series of the test substance (first fill and renewal); Table S2.1–S2.3. Results for water quality parameter monitoring (temperature pH, salinity, dissolved oxygen, and inorganic nitrogen measured at the beginning, before renewal-new-and the end-old-of each 24-h exposure period, for each mixture per concentration); Table S3. Equations for LC_50_ calculation for the reference toxicant tests 1, 2, and 3 (LC_50_ = 1.250, 1.739 mg/L, and 1.409 mg/L).
