# Supplementary material for: Suitability and Sensitivity of Golden Grey Mullet Chelon auratus (Risso, 1810) as a Reference Fish Species for Ecotoxicity Tests in the Black Sea
Source: Toxics. 2022 Apr 28;10(5):222. doi: 10.3390/toxics10050222 (PMC9144157; doi:10.3390/toxics10050222)
Supplement: Supplementary file 1 [file toxics-10-00222-s001.zip › toxics-1668289-supplementary.pdf]

# Supplementary Materials: Suitability and Sensitivity of Golden Grey Mullet *Chelon auratus* (Risso, 1810) as a Reference Fish Species for Ecotoxicity Tests in the Black Sea

**Victor Niță, Magda Nenciu and Valentina Coatu**

**Table S1.** Dilution series of the test substance (first fill and renewal).

| **Flask** | **First fill** | | | | **Renewal** | | | |
|---|---|---|---|---|---|---|---|---|
| | **Reference toxicant stock solution volume (ml)** | **Total solution volume (mL)** | **Reference toxicant concentration (mg/L)** | **Replicate solution volume (mL)** | **Reference toxicant stock solution volume (ml)** | Total solution volume (mL) | Reference toxicant concentration (mg/L) | Replicate solution volume (mL) |
| C0 | 0 | 25,000 | 0 | 5,000 | 0 | 20,000 | 0 | 4,000 |
| C1 | 5 | 25,000 | 0.2 | 5,000 | 4 | 20,000 | 0.2 | 4,000 |
| C2 | 10 | 25,000 | 0.4 | 5,000 | 8 | 20,000 | 0.4 | 4,000 |
| C3 | 20 | 25,000 | 0.8 | 5,000 | 16 | 20,000 | 0.8 | 4,000 |
| C4 | 40 | 25,000 | 1.6 | 5,000 | 32 | 20,000 | 1.6 | 4,000 |
| C5 | 80 | 25,000 | 3.2 | 5,000 | 64 | 20,000 | 3.2 | 4,000 |

**Table S2. 1-3.** Results of water quality parameter monitoring (temperature pH, salinity, dissolved oxygen and inorganic nitrogen measured at the beginning, before renewal - new - and the end - old - of each 24 h exposure period, for each mixture per concentration).

**Table S2.1. Run 1.**

| | **Variable** | **Treatments (mg/L)** | | | | | |
|---|---|---|---|---|---|---|---|
| | | **Control** | **1.25** | **2.5** | **5** | **10** | **20** |
| **0 h Experiment Start (new)** | Temperature (°C) | 21.8±0.52 | 21.28±0.36 | 20.54±0.19 | 20.94±0.90 | 21.5±1.25 | 21.14±1.19 |
| | Salinity (PSU) | 15.05±0.19 | 15.03±0.09 | 15.29±0.18 | 15.54±0.31 | 15.61±0.49 | 15.62±0.28 |
| | pH | 7.88±0.03 | 7.84±0.02 | 7.82±0.02 | 7.79±0.02 | 7.78±0.02 | 7.72±0.01 |
| | D.O. (mg/L) | 8.71±0.14 | 8.71±0.10 | 8.96±0.09 | 8.78±0.24 | 8.57±0.13 | 8.70±0.08 |
| | Oxygen saturation (%) | 108.36±1.27 | 106.72±1.37 | 108.48±0.43 | 107.94±1.35 | 105.66±0.88 | 107.32±2.15 |
| | $NO_2$ (µM) | 0.96 | 0.96 | 0.96 | 0.96 | 0.96 | 0.96 |
| | $NO_3$ (µM) | 34.02 | 34.02 | 34.02 | 34.02 | 34.02 | 34.02 |
| | $NH_4$ (µM) | 7.52 | 7.52 | 7.52 | 7.52 | 7.52 | 7.52 |
| | Temperature (°C) | 21.48±0.25 | 22.12±0.31 | 22.04±0.34 | 22.30±0.50 | 22.42±0.36 | 22.62±0.66 |
| | Salinity (PSU) | 15.13±0.13 | 14.98±0.09 | 15.05±0.17 | 15.05±0.14 | 15.06±0.14 | 15.08±0.23 |
| | pH | 7.45±0.08 | 7.49±0.07 | 7.54±0.03 | 7.60±0.04 | 7.65±0.06 | 7.71±0.03 |
| | D.O. (mg/L) | 2.64±0.87 | 3.87±1.13 | 5.25±0.23 | 6.27±0.48 | 6.21±1.06 | 7.91±0.21 |

| | | | | | | | |
|---|---|---|---|---|---|---|---|
| **24 h (old)** | Oxygen saturation (%) | 32.62±10.65 | 48.48±14.16 | 65.52±2.62 | 78.66±5.49 | 78.12±13.38 | 99.86±2.04 |
| | $NO_2$ (µM) | 1.07±0.18 | 0.95±0.05 | 1.17±0.08 | 2.24±1.05 | 3.98±0.31 | 3.38±0.07 |
| | $NO_3$ (µM) | - | - | - | 18.32±3.04 | 14.68±3.08 | 9.40±1.08 |
| | $NH_4$ (µM) | 80.61±10.77 | 71.96±4.77 | 73.70±11.80 | 75.41±10.44 | 96.44±18.34 | 74.07±9.81 |
| **24 h (new)** | Temperature (°C) | 20.92±0.36 | 20.94±0.15 | - | - | - | - |
| | Salinity (PSU) | 15.50±0.37 | 15.46±0.05 | - | - | - | - |
| | pH | 7.86±0.03 | 7.83±0.01 | - | - | - | - |
| | D.O. (mg/L) | 8.44±0.05 | 8.68±0.17 | - | - | - | - |
| | Oxygen saturation (%) | 103.54±1.04 | 106.42±1.77 | - | - | - | - |
| | $NO_2$ (µM) | 0.96 | 0.96 | - | - | - | - |
| | $NO_3$ (µM) | 34.02 | 34.02 | - | - | - | - |
| | $NH_4$ (µM) | 7.52 | 7.52 | - | - | - | - |
| **48 h (old)** | Temperature (°C) | 20.36±0.34 | 20.40±0.32 | - | - | - | - |
| | Salinity (PSU) | 15.20±0.04 | 15.17±0.05 | - | - | - | - |
| | pH | 7.37±0.04 | 7.41±0.04 | - | - | - | - |
| | D.O. (mg/L) | 3.17±1.16 | 4.17±1.01 | - | - | - | - |
| | Oxygen saturation (%) | 38.30±14.03 | 50.58±12.13 | - | - | - | - |
| | $NO_2$ (µM) | 8.28±2.02 | 4.42±0.74 | - | - | - | - |
| | $NO_3$ (µM) | 19.77±2.15 | 19.08±1.66 | - | - | - | - |
| | $NH_4$ (µM) | 144.47±3.90 | 143.04±7.15 | - | - | - | - |
| **48 h (new)** | Temperature (°C) | 20.94±0.13 | 21.04±0.05 | - | - | - | - |
| | Salinity (PSU) | 15.35±0.11 | 15.41±0.07 | - | - | - | - |
| | pH | 7.78±0.02 | 7.77±0.02 | - | - | - | - |
| | D.O. (mg/L) | 8.68±0.08 | 8.68±0.06 | - | - | - | - |
| | Oxygen saturation (%) | 106.38±0.90 | 106.62±0.86 | - | - | - | - |
| | $NO_2$ (µM) | 0.96 | 0.96 | - | - | - | - |
| | $NO_3$ (µM) | 34.02 | 34.02 | - | - | - | - |
| | $NH_4$ (µM) | 7.52 | 7.52 | - | - | - | - |
| **72 h (old)** | Temperature (°C) | 20.32±0.23 | 20.76±0.09 | - | - | - | - |
| | Salinity (PSU) | 15.36±0.05 | 15.34±0.09 | - | - | - | - |
| | pH | 7.39±0.04 | 7.41±0.03 | - | - | - | - |
| | D.O. (mg/L) | 4.49±0.97 | 4.98±0.81 | - | - | - | - |
| | Oxygen saturation (%) | 54.44±11.99 | 60.78±9.87 | - | - | - | - |
| | $NO_2$ (µM) | 7.57±1.23 | 6.33±1.59 | - | - | - | - |
| | $NO_3$ (µM) | 15.77±2.35 | 15.49±1.89 | - | - | - | - |
| | $NH_4$ (µM) | 154.69±2.54 | 162.00±3.72 | - | - | - | - |
| **72 h (new)** | Temperature (°C) | 20.04±0.26 | 20.22±0.25 | - | - | - | - |
| | Salinity (PSU) | 15.32±0.07 | 15.35±0.05 | - | - | - | - |
| | pH | 7.67±0.24 | 7.77±0.02 | - | - | - | - |
| | D.O. (mg/L) | 8.87±0.12 | 8.92±0.11 | - | - | - | - |
| | Oxygen saturation (%) | 106.90±1.16 | 107.90±0.93 | - | - | - | - |
| | $NO_2$ (µM) | 0.96 | 0.96 | - | - | - | - |
| | $NO_3$ (µM) | 34.02 | 34.02 | - | - | - | - |
| | $NH_4$ (µM) | 7.52 | 7.52 | - | - | - | - |
| | Temperature (°C) | 21.46±0.25 | 21.10±0.19 | - | - | - | - |

| | | | | | | | |
|---|---|---|---|---|---|---|---|
| **(96 h) Experiment end (old)** | Salinity (PSU) | 15.09±0.06 | 15.06±0.10 | - | - | - | - |
| | pH | 7.40±0.05 | 7.40±0.05 | - | - | - | - |
| | D.O. (mg/L) | 4.59±1.10 | 4.21±0.90 | - | - | - | - |
| | Oxygen saturation (%) | 56.70±13.43 | 51.70±11.00 | - | - | - | - |
| | $NO_2$ (μM) | 7.32±2.09 | 2.25±1.02 | - | - | - | - |
| | $NO_3$ (μM) | 12.80±3.30 | 11.42±2.11 | - | - | - | - |
| | $NH_4$ (μM) | 147.64±6.12 | 151.46±6.70 | - | - | - | - |

**Table S2.2. Run 2.**

| | | **Treatments (mg/L)** | | | | | |
|---|---|---|---|---|---|---|---|
| | **Variable** | **Control** | **0.2** | **0.4** | **0.8** | **1.6** | **3.2** |
| **0 h Experiment Start (new)** | Temperature (°C) | 19.5±0.00 | 19.5±0.00 | 19.46±0.09 | 19.64±0.19 | 19.24±0.13 | 19.54±0.11 |
| | Salinity (PSU) | 15.95±0.11 | 15.97±0.07 | 16.04±0.06 | 15.90±0.08 | 15.92±0.11 | 15.94±0.11 |
| | pH | 8.1±0.00 | 8.18±0.02 | 8.14±0.01 | 8.14±0.01 | 8.09±0.00 | 8.14±0.01 |
| | D.O. (mg/L) | 8.33±0.02 | 8.47±0.07 | 8.41±0.04 | 8.36±0.07 | 8.58±0.09 | 8.52±0.12 |
| | Oxygen saturation (%) | 99.67±0.28 | 101.38±0.82 | 100.62±0.33 | 100.39±0.05 | 102.12±0.97 | 102.01±1.34 |
| | $NO_2$ (μM) | 0.19 | 0.19 | 0.19 | 0.19 | 0.19 | 0.19 |
| | $NO_3$ (μM) | 3.23 | 3.23 | 3.23 | 3.23 | 3.23 | 3.23 |
| | $NH_4$ (μM) | 2.59 | 2.59 | 2.59 | 2.59 | 2.59 | 2.59 |
| **24 h (old)** | Temperature (°C) | 20.48±0.24 | 20.7±0.35 | 20.70±0.2 | 20.76±0.15 | 20.70±0.25 | - |
| | Salinity (PSU) | 16.10±0.1 | 16.03±0.16 | 15.94±0.08 | 16.02±0.12 | 16.10±0.11 | - |
| | pH | 7.63±0.05 | 7.61±0.02 | 7.57±0.04 | 7.56±0.07 | 7.48±0.10 | - |
| | D.O. (mg/L) | 7.26±0.42 | 6.65±0.48 | 6.75±0.39 | 6.69±0.67 | 6.83±0.39 | - |
| | Oxygen saturation (%) | 88.59±5.18 | 81.47±5.79 | 82.68±4.73 | 81.99±8.19 | 83.71±4.94 | - |
| | $NO_2$ (μM) | 0.3±0.09 | 0.23±0.03 | 0.27±0.02 | 0.26±0.03 | 0.28±0.02 | - |
| | $NO_3$ (μM) | 3.35±0.78 | 2.81±0.23 | 3.10±0.21 | 3.15±0.29 | 3.45±0.67 | - |
| | $NH_4$ (μM) | 21.67±5.19 | 22.54±3.56 | 20.51±2.57 | 23.42±3.76 | 28.09±4.00 | - |
| **24 h (new)** | Temperature (°C) | 19.24±0.21 | 19.36±0.27 | 19.68±0.19 | 19.58±0.41 | 19.50±0.32 | - |
| | Salinity (PSU) | 15.92±0.13 | 15.91±0.13 | 15.91±0.2 | 15.96±0.09 | 15.81±0.14 | - |
| | pH | 8.11±0.01 | 8.11±0.03 | 8.07±0.02 | 8.11±0.01 | 8.11±0.01 | - |
| | D.O. (mg/L) | 8.80±0.15 | 8.53±0.14 | 8.63±0.08 | 8.34±0.17 | 8.45±0.21 | - |
| | Oxygen saturation (%) | 104.71±1.35 | 101.79±1.72 | 103.63±0.95 | 100.00±1.50 | 101.07±2.18 | - |
| | $NO_2$ (μM) | 0.19 | 0.19 | 0.19 | 0.19 | 0.19 | - |
| | $NO_3$ (μM) | 3.23 | 3.23 | 3.23 | 3.23 | 3.23 | - |
| | $NH_4$ (μM) | 2.59 | 2.59 | 2.59 | 2.59 | 2.59 | - |
| **48 h (old)** | Temperature (°C) | 20.20±0.16 | 20.58±0.13 | 20.26±0.21 | 20.34±0.15 | 20.66±0.19 | - |
| | Salinity (psu) | 15.94±0.06 | 15.99±0.09 | 16.02±0.14 | 16.00±0.10 | 16.17±0.11 | - |
| | pH | 7.22±0.04 | 7.18±0.03 | 7.20±0.04 | 7.18±0.01 | 7.16±0.04 | - |
| | D.O. (mg/L) | 7.73±0.16 | 6.93±1.01 | 7.49±0.36 | 7.29±0.12 | 7.06±0.41 | - |
| | Oxygen saturation (%) | 93.78±2.01 | 84.73±12.21 | 90.97±4.41 | 88.69±1.41 | 86.48±4.98 | - |
| | $NO_2$ (μM) | 0.22±0.02 | 0.21±0.01 | 0.20±0.01 | 0.05±0.02 | 0.22±0.04 | - |
| | $NO_3$ (μM) | 2.02±0.14 | 2.17±0.16 | 2.15±0.32 | 2.22±0.2 | 2.08±0.09 | - |
| | $NH_4$ (μM) | 24.23±3.52 | 25.24±4.34 | 21.00±3.16 | 31.16±5.27 | 37.86±5.47 | - |

| | | | | | | | |
|---|---|---|---|---|---|---|---|
| **48 h (new)** | Temperature (°C) | 19.44±0.24 | 19.66±0.29 | 19.10±0.35 | 19.38±0.46 | 17.20±0.2 | - |
| | Salinity (PSU) | 15.97±0.06 | 15.97±0.04 | 15.92±0.08 | 15.96±0.13 | 13.82±0.04 | - |
| | pH | 7.58±0.04 | 7.59±0.04 | 7.57±0.02 | 7.56±0.01 | 6.69±0.02 | - |
| | D.O. (mg/L) | 8.48±0.07 | 8.32±0.08 | 8.49±0.04 | 8.44±0.14 | 6.83±0.05 | - |
| | Oxygen saturation (%) | 101.34±0.83 | 99.86±0.43 | 100.83±0.26 | 100.72±0.79 | 82.00±8.38 | - |
| | $NO_2$ (μM) | 0.19 | 0.19 | 0.19 | 0.19 | 0.19 | - |
| | $NO_3$ (μM) | 3.23 | 3.23 | 3.23 | 3.23 | 3.23 | - |
| | $NH_4$ (μM) | 2.59 | 2.59 | 2.59 | 2.59 | 2.59 | - |
| **72 h (old)** | Temperature (°C) | 20.72±0.27 | 20.9±0.17 | 20.80±0.33 | 20.76±0.05 | 21.27±0.23 | - |
| | Salinity (PSU) | 15.71±0.17 | 15.73±0.14 | 15.63±0.13 | 15.67±0.06 | 15.70±0.14 | - |
| | pH | 7.24±0.03 | 7.23±0.03 | 7.21±0.02 | 7.21±0.03 | 7.20±0.06 | - |
| | D.O. (mg/L) | 7.36±0.43 | 7.26±0.19 | 7.25±0.22 | 7.12±0.28 | 6.80±0.86 | - |
| | Oxygen saturation (%) | 90.01±5.08 | 89.07±2.16 | 88.80±2.30 | 87.15±3.43 | 83.99±10.38 | - |
| | $NO_2$ (μM) | 0.12±0.02 | 0.12±0.01 | 0.10±0.00 | 0.09±0.01 | 0.08±0.02 | - |
| | $NO_3$ (μM) | 2.53±0.06 | 2.62±0.19 | 2.74±0.08 | 2.67±0.23 | 2.87±0.16 | - |
| | $NH_4$ (μM) | 27.56±4.01 | 28.78±4.22 | 25.53±3.69 | 31.52±4.16 | 35.07±3.33 | - |
| **72 h (new)** | Temperature (°C) | 19.50±0.18 | 19.86±0.19 | 19.66±0.05 | 19.78±0.40 | 19.52±0.15 | - |
| | Salinity (PSU) | 15.44±0.26 | 15.51±0.06 | 15.52±0.06 | 15.52±0.15 | 15.58±0.12 | - |
| | pH | 7.52±0.01 | 7.51±0.01 | 7.49±0.01 | 7.46±0.00 | 7.45±0.01 | - |
| | D.O. (mg/L) | 8.34±0.03 | 8.20±0.06 | 8.25±0.05 | 8.21±0.10 | 8.28±0.06 | - |
| | Oxygen saturation (%) | 99.55±0.27 | 98.56±0.69 | 98.74±0.49 | 98.53±0.46 | 98.96±0.46 | - |
| | $NO_2$ (μM) | 0.19 | 0.19 | 0.19 | 0.19 | 0.19 | - |
| | $NO_3$ (μM) | 3.23 | 3.23 | 3.23 | 3.23 | 3.23 | - |
| | $NH_4$ (μM) | 2.59 | 2.59 | 2.59 | 2.59 | 2.59 | - |
| **(96 h) Experiment end (old)** | Temperature (°C) | 20.56±0.11 | 21.06±0.42 | 21.22±0.16 | 21.52±0.31 | 21.54±0.30 | - |
| | Salinity (PSU) | 16.13±0.15 | 16.09±0.15 | 16.17±0.14 | 16.10±0.09 | 16.25±0.17 | - |
| | pH | 7.28±0.08 | 7.12±0.15 | 7.22±0.22 | 7.13±0.10 | 7.22±0.09 | - |
| | D.O. (mg/L) | 7.31±0.48 | 4.24±2.59 | 6.42±1.01 | 5.87±1.27 | 6.63±1.15 | - |
| | Oxygen saturation (%) | 89.40±5.90 | 52.46±32.27 | 79.55±12.57 | 73.06±15.58 | 82.59±14.36 | - |
| | $NO_2$ (μM) | 0.17±0.02 | 0.15±0.02 | 0.16±0.03 | 0.14±0.01 | 0.14±0.02 | - |
| | $NO_3$ (μM) | 2.07±0.15 | 2.23±0.13 | 2.31±0.46 | 1.93±0.21 | 2.06±0.15 | - |
| | $NH_4$ (μM) | 25.11±6.27 | 30.61±5.38 | 27.21±3.51 | 33.02±4.52 | 32.99±5.88 | - |

**Table S2.3. Run 3.**

| | Variable | Treatments (mg/L) | | | | | |
|---|---|---|---|---|---|---|---|
| | | Control | 0.2 | 0.4 | 0.8 | 1.6 | 3.2 |
| **0 h Experiment Start (new)** | Temperature (°C) | 21.18±0.04 | 21.10±0.00 | 21.00±0.0 | 20.90±0.17 | 20.86±0.09 | 20.78±0.04 |
| | Salinity (PSU) | 15.96±0.05 | 16.10±0.19 | 16.08±0.20 | 16.03±0.19 | 15.98±0.19 | 16.00±0.13 |
| | pH | 7.78±0.02 | 7.79±0.02 | 7.74±0.02 | 7.66±0.01 | 7.62±0.02 | 7.56±0.01 |
| | D.O. (mg/L) | 8.65±0.03 | 8.76±0.0 | 8.74±0.08 | 8.67±0.05 | 8.60±0.03 | 8.63±0.07 |
| | Oxygen saturation (%) | 97.32±0.38 | 98.38±0.92 | 98.09±0.82 | 97.15±0.21 | 96.24±0.21 | 96.33±074 |
| | $NO_2$ (µM) | 0.29 | 0.29 | 0.29 | 0.29 | 0.29 | 0.29 |
| | $NO_3$ (µM) | 12.44 | 12.44 | 12.44 | 12.44 | 12.44 | 12.44 |
| | $NH_4$ (µM) | 0.92 | 0.92 | 0.92 | 0.92 | 0.92 | 0.92 |
| **24 h (old)** | Temperature (°C) | 19.50±0.19 | 19.56±0.21 | 19.54±0.21 | 19.40±0.16 | 19.52±0.11 | 19.64±0.28 |
| | Salinity (PSU) | 16.34±0.10 | 16.36±0.15 | 16.39±0.12 | 16.43±0.09 | 16.37±0.03 | 16.41±0.15 |
| | pH | 7.32±0.04 | 7.33±0.04 | 7.30±0.05 | 7.30±0.04 | 7.30±0.02 | 7.28±0.13 |
| | D.O. (mg/L) | 8.50±0.07 | 8.41±0.17 | 8.32±0.23 | 8.51±0.13 | 8.34±0.22 | 8.20±0.36 |
| | Oxygen saturation (%) | 92.57±0.96 | 91.74±1.74 | 90.69±2.34 | 92.52±1.22 | 90.93±2.33 | 89.49±3.66 |
| | $NO_2$ (µM) | 0.20±0.01 | 0.21±0.03 | 0.22±0.01 | 0.25±0.03 | 0.26±0.02 | 0.61±0.11 |
| | $NO_3$ (µM) | 13.62±0.52 | 14.72±0.78 | 14.64±0.71 | 14.67±0.68 | 15.65±0.61 | 19.73±0.12 |
| | $NH_4$ (µM) | 6.83±0.60 | 6.85±1.56 | 8.92±1.22 | 8.33±0.81 | 10.82±1.12 | 11.83±2.31 |
| **24 h (new)** | Temperature (°C) | 19.66±0.09 | 20.14±0.05 | 20.20±0.07 | 20.18±0.04 | 20.22±0.04 | - |
| | Salinity (PSU) | 16.38±0.09 | 16.48±0.03 | 16.46±0.08 | 16.42±0.02 | 16.47±0.06 | - |
| | pH | 7.53±0.01 | 7.64±0.02 | 7.58±0.01 | 7.56±0.02 | 7.52±0.01 | - |
| | D.O. (mg/L) | 8.49±0.04 | 8.91±0.12 | 8.82±0.09 | 8.74±0.03 | 8.73±0.08 | - |
| | Oxygen saturation (%) | 92.77±0.42 | 98.21±1.34 | 97.37±0.89 | 96.49±0.24 | 96.38±0.81 | - |
| | $NO_2$ (µM) | 0.29 | 0.29 | 0.29 | 0.29 | 0.29 | - |
| | $NO_3$ (µM) | 12.44 | 12.44 | 12.44 | 12.44 | 12.44 | - |
| | $NH_4$ (µM) | 0.92 | 0.92 | 0.92 | 0.92 | 0.92 | - |
| **48 h (old)** | Temperature (°C) | 19.38±0.24 | 19.24±0.15 | 19.30±0.14 | 19.2±0.19 | 19.48±0.19 | - |
| | Salinity (PSU) | 16.30±0.10 | 16.33±0.07 | 16.43±0.11 | 16.37±0.06 | 16.26±0.10 | - |
| | pH | 7.30±0.03 | 7.29±0.02 | 7.26±0.01 | 7.25±0.03 | 7.26±0.02 | - |
| | D.O. (mg/L) | 8.88±0.13 | 8.81±0.25 | 8.87±0.10 | 8.86±0.13 | 8.81±0.19 | - |
| | Oxygen saturation (%) | 96.48±1.20 | 95.47±2.79 | 96.20±0.78 | 105.68±1.15 | 105.53±2.03 | - |
| | $NO_2$ (µM) | 0.25±0.04 | 0.25±0.05 | 0.24±0.02 | 0.25±0.02 | 0.27±0.03 | - |
| | $NO_3$ (µM) | 13.09±0.33 | 13.30±1.14 | 12.76±0.99 | 13.31±0.62 | 13.52±1.05 | - |
| | $NH_4$ (µM) | 6.47±1.01 | 8.25±0.85 | 9.76±0.79 | 9.83±1.25 | 13.83±2.69 | - |

| | | | | | | | |
|---|---|---|---|---|---|---|---|
| **48 h (new)** | Temperature (°C) | 19.74±0.05 | 20±0.07 | 20±0.07 | 20.08±0.04 | 20.08±0.04 | - |
| | Salinity (PSU) | 16.37±0.09 | 16.41±0.07 | 16.42±0.06 | 16.41±0.10 | 16.39±0.10 | - |
| | pH | 7.41±0.01 | 7.46±0.01 | 7.43±0.02 | 7.41±0.01 | 7.38±0.01 | - |
| | D.O. (mg/L) | 8.38±0.03 | 8.96±0.11 | 8.85±0.05 | 8.85±0.10 | 8.78±0.09 | - |
| | Oxygen saturation (%) | 91.69±0.38 | 98.61±0.93 | 107.22±0.60 | 107.40±1.20 | 106.60±0.98 | - |
| | $NO_2$ (μM) | 0.29 | 0.29 | 0.29 | 0.29 | 0.29 | - |
| | $NO_3$ (μM) | 12.44 | 12.44 | 12.44 | 12.44 | 12.44 | - |
| | $NH_4$ (μM) | 0.92 | 0.92 | 0.92 | 0.92 | 0.92 | - |
| **72 h (old)** | Temperature (°C) | 19.4±0.27 | 19.18±0.08 | 19.24±0.19 | 19.32±0.23 | 19.70±0.28 | - |
| | Salinity (PSU) | 16.32±0.10 | 16.30±0.10 | 16.31±0.15 | 16.43±0.19 | 16.39±0.04 | - |
| | pH | 7.25±0.03 | 7.26±0.01 | 7.23±0.02 | 7.22±0.02 | 7.23±0.03 | - |
| | D.O. (mg/L) | 8.71±0.12 | 8.70±0.21 | 8.54±0.29 | 8.65±0.34 | 8.80±.15 | - |
| | Oxygen saturation (%) | 104.31±0.93 | 103.72±2.46 | 101.88±3.37 | 103.45±3.52 | 106.03±2.28 | - |
| | $NO_2$ (μM) | 0.22±0.02 | 0.23±0.04 | 0.46±0.05 | 0.62±0.07 | 0.28±0.14 | - |
| | $NO_3$ (μM) | 10.49±1.03 | 11.49±0.61 | 10.30±0.51 | 10.67±0.42 | 8.52±4.54 | - |
| | $NH_4$ (μM) | 7.00±0.98 | 7.98±0.80 | 9.88±0.57 | 9.83±0.36 | 7.47±4.30 | - |
| **72 h (new)** | Temperature (°C) | 19.68±0.08 | 20.06 ±0.05 | 20.08±0.04 | 20.08±0.04 | 20.03 ±0.12 | - |
| | Salinity (PSU) | 16.34±0.05 | 16.40 ±0.09 | 16.40±0.09 | 16.40±0.06 | 16.45±0.08 | - |
| | pH | 7.34±0.01 | 7.40 ±0.02 | 7.36±0.01 | 7.32±0.02 | 7.30 ±0.02 | - |
| | D.O. (mg/L) | 7.96±0.03 | 8.67±0.09 | 8.62±0.08 | 8.62±0.12 | 8.62±0.04 | - |
| | Oxygen saturation (%) | 97.20±3.06 | 105.14±1.02 | 104.54±0.94 | 104.61±1.35 | 104.57±0.36 | - |
| | $NO_2$ (μM) | 0.29 | 0.29 | 0.29 | 0.29 | 0.29 | - |
| | $NO_3$ (μM) | 12.44 | 12.44 | 12.44 | 12.44 | 12.44 | - |
| | $NH_4$ (μM) | 0.92 | 0.92 | 0.92 | 0.92 | 0.92 | - |
| **(96 h) Experiment end (old)** | Temperature (°C) | 19.06±0.21 | 19.18±0.22 | 19.12±0.15 | 19.08±0.15 | 19.6±0.36 | - |
| | Salinity (PSU) | 16.34±0.10 | 16.34±0.17 | 16.45±0.08 | 16.42±0.07 | 16.38±0.25 | - |
| | pH | 7.38±0.02 | 7.35±0.03 | 7.35±0.04 | 7.39±0.02 | 7.39±0.01 | - |
| | D.O. (mg/L) | 8.77±0.12 | 8.36±0.50 | 8.56±0.29 | 8.72±0.08 | 8.82±0.05 | - |
| | Oxygen saturation (%) | 104.33±1.36 | 99.67±5.86 | 102.05±3.24 | 103.86±0.69 | 106.51±1.27 | - |
| | $NO_2$ (μM) | 0.29±0.02 | 0.27±0.02 | 0.27±0.00 | 0.28±0.01 | 0.23±0.12 | - |
| | $NO_3$ (μM) | 6.63±0.41 | 6.96±0.20 | 6.81±0.30 | 6.82±0.42 | 5.79±3.04 | - |
| | $NH_4$ (μM) | 6.91±0.68 | 8.66±1.15 | 9.40±0.68 | 10.36±0.41 | 6.21±3.80 | - |

**Table S3.** Equations for $LC_{50}$ calculation for the reference toxicant tests 1, 2 and 3 ($LC_{50}$ = 1.250, 1.739 mg/L, 1.409 mg/L).

| Parameter | Value |
|---|---|
| $LC_{50}$ | 1.25 |
| Equation | $Y = -0.0981 + \frac{100.0916 + (-0.0981)}{1 + (\frac{X}{1.25})^{-8.1493}}$ |
| Equation Form | $Y = Min + \frac{Max - Min}{1 + (\frac{X}{LC50})^{Hill\ coefficient}}$ |

| Parameter | Value |
|---|---|
| $LC_{50}$ | 1.7392 |
| Equation | $Y = -0.0091 + \frac{100.2008 + (-0.0091)}{1 + (\frac{X}{1.7392})^{-10.1878}}$ |
| Equation Form | $Y = Min + \frac{Max - Min}{1 + (\frac{X}{LC50})^{Hill\ coefficient}}$ |

| Parameter | Value |
|---|---|
| $LC_{50}$ | 1.4095 |
| Equation | $Y = -0.0113 + \frac{100.0014 + (-0.0113)}{1 + (\frac{X}{1.4095})^{-13.6797}}$ |
| Equation Form | $Y = Min + \frac{Max - Min}{1 + (\frac{X}{LC50})^{Hill\ coefficient}}$ |
